# The effect of *LTA* gene polymorphisms on cancer risk: an updated systematic review and meta- analysis

**DOI:** 10.1042/BSR20192320

**Published:** 2020-05-28

**Authors:** Jingdong Li, Yaxuan Wang, Xueliang Chang, Zhenwei Han

**Affiliations:** Department of Urology, The Second Hospital of Hebei Medical University, Shijiazhuang 050000, China

**Keywords:** tumour necrosis factors, cancer, single nucleotide polymorphisms, meta analysis

## Abstract

**Purpose:** To provide a comprehensive account of the association of five Lymphotoxin-α (*LTA*) gene polymorphisms (rs1041981, rs2229094, rs2239704, rs746868, rs909253) with susceptibility to cancer.

**Methods:** A literature search for eligible candidate gene studies published before 28 February 2020 was conducted in the PubMed, Medline, Google Scholar and Web of Science. The following combinations of main keywords were used: (LTA OR Lymphotoxin alpha OR TNF-β OR tumor necrosis factor-beta) AND (polymorphism OR mutation OR variation OR SNP OR genotype) AND (cancer OR tumor OR neoplasm OR malignancy OR carcinoma OR adenocarcinoma). Potential sources of heterogeneity were sought out via subgroup and sensitivity analysis, and publication bias were estimated.

**Results:** Overall, a total of 24 articles with 24577 cases and 33351 controls for five polymorphisms of *LTA* gene were enrolled. We identified that rs2239704 was associated with a reduced risk of cancer. While for other polymorphisms, the results showed no significant association with cancer risk. In the stratified analysis of rs1041981, we found that Asians might have less susceptibility to cancer. At the same time, we found that rs2239704 was negatively correlated with non-Hodgkin lymphoma (NHL). While, for rs909253, an increased risk of cancer for Caucasians and HCC susceptibility were uncovered in the stratified analysis of by ethnicity and cancer type.

**Conclusion:** *LTA* rs2239704 polymorphism is inversely associated with the risk of cancer. *LTA* rs1041981 polymorphism is negatively associated with cancer risk in Asia. While, *LTA* rs909253 polymorphism is a risk factor for HCC in Caucasian population.

## Introduction

Increasing studies have demonstrated that a number of proinflammatory cytokines could be associated with the development of cancer [1,2]. Lymphotoxin-α (LTA) is the predominant member of the tumor necrosis factor (TNF) ligand family, which responds to immune and inflammatory reaction and plays an important role in the pathogenesis of cancer [3,4]. The human *LTA* gene is located on the short arm of chromosome 6 (6p21.3) [5]. The presence of single nucleic polymorphism (SNP) may affect cytokine expression level, which might be an important mediator of cancer [6,7]. SNP rs1041981 is a mutation of *LTA* gene at the 804 (C/A) position of exon 3 in codon 26, causing the amino acid threonine to be asparagine, which may be related to the transcriptional regulation of *LTA*, then activate the lymphocytes and induce apoptosis [8]. While, SNP rs909253 is a mutation of *LTA* gene at 252 (A/G) position in intron 1, which may lead to increase in the transcriptional activities of *LTA* [1]. In addition, SNP rs2239704, rs746868 and rs2229094 are associated with the *LTA* expression, which may affect subsequent inflammatory responses and immunomodulatory diseases, including cancers [9,10].

There are ample evidences that have demonstrated the association between *LTA* polymorphisms and cancer [11–34]. However, these results are inconsistent and even contradictory, which might be due to the heterogeneity within cancer types, ethnicities, source of control, Hardy–Weinberg equilibrium (HWE), small sample sizes and so on. Huang et al. reported a meta-analysis about this topic, and they found that the *LTA* rs1041981, rs2239704 and rs2229094 polymorphisms were associated with the increased risk of cancers [35]. However, based on the current studies, we found that more studies were negatively correlated with *LTA* polymorphisms and cancer [11,13–15,18–24,27,28,31,32]. Therefore, we conducted the current updated systematic review and meta-analysis to accurately determine the association between genetic variation of *LTA* gene and cancer susceptibility.

## Materials and methods

### Literature search

We conducted a systematic literature search on PubMed, Medline, Embase, Google Scholar and Web of Science to retrieve all eligible publications on the association between *LTA* polymorphisms and the risk of cancer (up to 28 February 2020) with the following keywords: (LTA OR Lymphotoxin alpha OR TNF-β OR tumor necrosis factor-beta) AND (polymorphism OR mutation OR variation OR SNP OR genotype) AND (cancer OR tumor OR neoplasm OR malignancy OR carcinoma OR adenocarcinoma). The language of enrolled studies was restricted to English. After carefully screening, five polymorphisms were left for further investigation.

### Inclusion and exclusion criteria

Articles enrolled in our meta-analysis satisfied the following inclusion criteria: (1) case–control studies that evaluated the association between *LTA* polymorphisms and cancer risk; (2) publications focusing on population genetic polymorphisms; (3) articles with sufficient genotype data to assess odds ratios (ORs) and the corresponding 95% CIs; (4) blood sample only for SNP analysis; (5) the control subjects satisfied HWE. The major exclusion criteria were: (1) case-only studies, case reports or reviews; (2) studies without raw data for the LTA genotype.

### Data extraction

Two investigators (Jingdong Li and Yaxuan Wang) independently extracted the data from each study. All the case–control studies satisfied the inclusion criteria and consensus for any controversy was achieved. The data from the eligible articles comprise the first author’s name, year of publication, ethnicity, source of control, cancer type and numbers of cases and controls in *LTA* genotypes. Ethnicity was categorized as ‘Asian’, ‘Caucasian’, and ‘Mixed’.

### Statistical analysis

The risk between the *LTA* polymorphisms and cancer was evaluated using summary ORs and the corresponding 95% CIs in allelic (B vs. A), dominant (BA + BB vs. AA), and recessive (BB vs. BA + AA) models (A: wild allele; B: mutated allele). The Cochrane’s Q-statistic test was used to assess the heterogeneity between studies, and the inconsistency was quantified with the *I^2^* statistic. The substantial heterogeneity was considered significant when *I^2^* > 50% or PQ ≤ 0.1, then, a random-effects model was used; otherwise, the fixed-effects model was applied. Subgroup meta-analysis were performed by cancer type, ethnicity, genotyping, HWE and the source of control. We also conducted sensitivity analysis to assess stability of the results by omitting one study each time to exclude studies. HWE was estimated by the asymptotic test, and deviation was considered when *P*<0.05. The potential publication bias of the eligible studies was evaluated by Begg’s and Egger’s regression test quantitatively. Trial sequential analysis (TSA) was performed as described by Xie et al. [36]. The required information size was calculated after adopting a level of significance of 5% for type I error and of 30% for type II error. The data was analyzed using the Stata 14.0 software (version 14.0; State Corporation, College Station, Texas, U.S.A.). A two-tailed *P*<0.05 was considered statistically significant.

## Results

### Main characteristics of the enrolled studies

**Figure 1:**
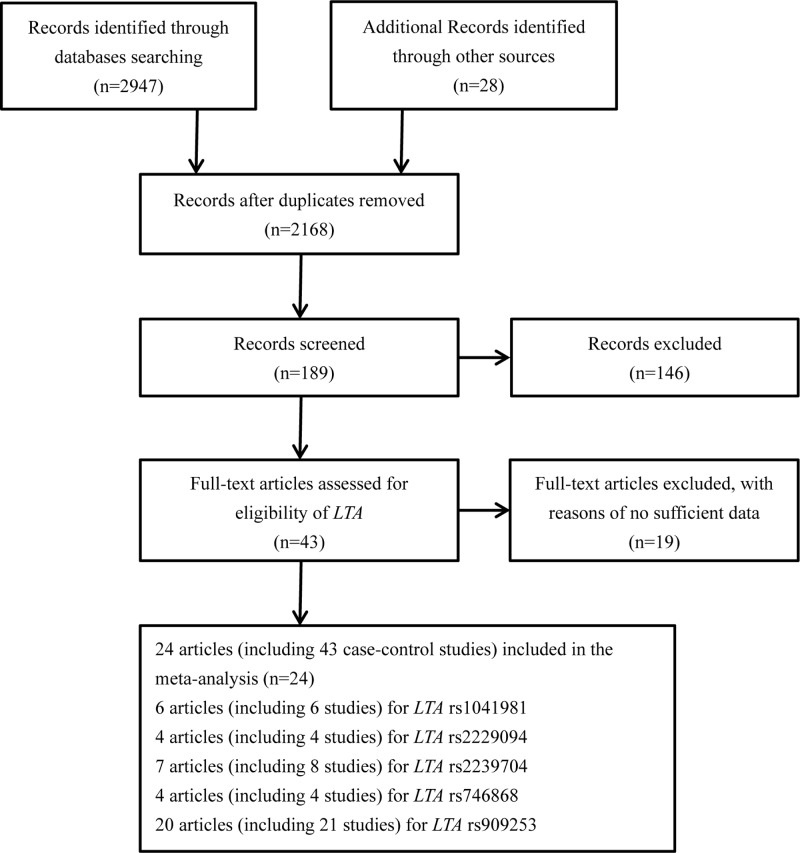
Flow chart of studies selection process for *LTA* gene polymorphisms

The study selection processes were presented in Figure 1. For polymorphisms of *LTA* gene (rs1041981, rs2229094, rs2239704, rs746868, rs909253), a total of 24 articles (including 43 case–control studies) with 24577 cases and 33351 controls met the inclusion criteria [11–34]. Sixteen of these studies were performed in Asians, 17 studies were performed in Caucasians, 10 studies in Africans and the others were in mixed ethnic groups (including at least one race). Controls of 30 studies were population-based controls and 13 studies were hospital-based controls. All studies were in compliance with HWE except for two studies [30,32]. Table 1 shows the characteristics of all the eligible studies and genotype frequency distributions of the five *LTA* polymorphisms included in our meta-analysis. Newcastle–Ottawa scale (NOS) was used to evaluate the quality of the enrolled studies, as shown in Table 2.

**Table 1:** Characteristics of the enrolled studies

SNP	First author	Year	Ethnicity	Source of control	Cancer type	Case			Control			HWE
						AA	AB	BB	AA	AB	BB	
rs1041981	Abbas	2010	Caucasian	PB	OC	1498	1317	332	2481	2399	607	Y
	Castro	2009	Caucasian	PB	OC	154	456	341	337	813	557	Y
	Lee	2004	Asian	PB	GC	109	156	63	74	132	47	Y
	Niwa	2005	Asian	HB	OC	60	59	12	107	165	48	Y
	Niwa	2007	Asian	HB	OC	51	43	16	71	114	35	Y
	Sainz	2012	Caucasian	PB	OC	833	729	198	794	760	173	Y
rs2229094	Abbas	2010	Caucasian	PB	OC	1686	1199	251	2965	2153	359	Y
	Madeleine	2011	Mixed	PB	OC	444	329	75	475	334	57	Y
	Mahajan	2008	Caucasian	PB	GC	206	74	21	247	150	18	Y
	Wang	2009	Mixed	PB	NHL	1043	751	148	978	702	124	Y
rs2239704	Cerhan	2008	Mixed	HB	NHL	169	217	55	170	225	79	Y
	Ennas	2008	Caucasian	PB	OC	14	17	7	36	53	23	Y
	Gu	2014	Asian	PB	NHL	33	50	10	82	96	25	Y
	Gu	2014	Asian	PB	NHL	30	21	13	82	100	47	Y
	Lan	2006	Mixed	PB	NHL	165	189	63	186	226	87	Y
	Mahajan	2008	Caucasian	PB	GC	85	138	76	105	223	85	Y
	Purdue	2007	Caucasian	HB	NHL	202	240	64	162	229	72	Y
	Wang	2009	Mixed	PB	NHL	697	754	241	599	714	256	Y
rs746868	Crusius	2008	Caucasian	PB	GC	151	205	72	398	545	181	Y
	Garcia-Gonzalez	2007	Caucasian	PB	GC	135	194	75	142	191	71	Y
	Gunter	2006	Mixed	HB	OC	85	107	27	76	102	27	Y
	Mahajan	2008	Caucasian	PB	GC	83	143	74	108	220	84	Y
rs909253	Cerhan	2008	Mixed	HB	NHL	179	208	53	207	217	51	Y
	Cheng	2015	Asian	HB	NHL	45	71	9	95	149	56	Y
	Crusius	2008	Caucasian	PB	GC	168	218	38	533	472	121	Y
	Ennas	2008	Caucasian	PB	OC	29	10	1	85	24	4	Y
	Garcia-Gonzalez	2007	Caucasian	PB	GC	238	127	39	222	154	28	Y
	Gu	2014	Asian	PB	NHL	42	39	11	69	98	36	Y
	Gu	2014	Asian	PB	NHL	27	29	8	104	97	28	Y
	Gunter	2006	Mixed	HB	OC	90	101	35	88	92	29	Y
	Jeng	2014	Asian	PB	HCC	46	65	39	98	42	10	Y
	Lakhanpal	2016	Asian	HB	OC	14	59	47	39	24	37	N
	Lan	2006	Mixed	PB	NHL	240	218	59	274	254	65	Y
	Lee	2004	Asian	PB	GC	112	152	64	77	131	46	Y
	Liu	2013	Asian	PB	NHL	111	151	29	95	149	56	Y
	Mahajan	2008	Caucasian	PB	GC	137	135	29	201	174	38	Y
	Mou	2015	Asian	HB	GC	105	75	14	57	48	28	N
	Niwa	2005	Asian	HB	OC	60	59	12	107	165	48	Y
	Niwa	2007	Asian	HB	OC	51	43	16	71	114	35	Y
	Purdue	2007	Caucasian	HB	NHL	205	265	68	198	233	63	Y
	Tsai	2017	Asian	PB	HCC	45	66	39	98	42	10	Y
	Wang	2009	Mixed	PB	NHL	778	857	262	788	766	219	Y
	Yri	2013	Caucasian	PB	NHL	157	247	76	394	479	136	Y

Abbreviations: GC, gastric cancer; HB, hospital-based; HCC, hepatocellular carcinoma; N, no; NHL, non-Hodgkin lymphoma; OC, other cancer; PB, population-based; Y, yes.

**Table 2:** Methodological quality of the enrolled studies according to the NOS

SNP	First author	Adequacy definition	Representativeness of the cases	Control selection	Control definition	Comparability cases/ controls	Exposure ascertainment	Same method ascertainment	Non-response rate
rs1041981	Abbas et al.	*	*	*	*	**	*	*	*
	Castro et al.	*	*	*	*	**	**	*	*
	Lee et al.	*	*	*	*	**	*	*	*
	Niwa et al.	*	*	NA	*	**	*	*	*
	Niwa et al.	*	*	NA	*	**	*	*	*
	Sainz et al.	*	*	*	*	**	*	*	*
rs2229094	Abbas et al.	*	*	*	*	**	*	*	*
	Madeleine et al.	*	*	*	*	**	*	*	*
	Mahajan et al.	*	*	*	*	**	*	*	*
	Wang et al.	*	*	*	*	**	*	*	*
rs2239704	Cerhan et al.	*	*	NA	*	**	*	*	*
	Ennas et al.	*	*	*	*	**	*	*	*
	Gu et al.	*	*	NA	*	**	*	*	*
	Gu et al.	*	*	NA	*	**	*	*	*
	Lan et al.	*	*	*	*	**	*	*	*
	Mahajan et al.	*	*	*	*	**	*	*	*
	Purdue et al.	*	*	NA	*	**	*	*	*
	Wang et al.	*	*	*	*	**	*	*	*
rs746868	Crusius et al.	*	*	NA	*	**	*	*	*
	Garcia-Gonzalez et al.	*	*	*	*	**	*	*	*
	Gunter et al.	*	*	NA	*	**	**	*	*
	Mahajan et al.	*	*	*	*	**	*	*	*
rs909253	Cerhan et al.	*	*	NA	*	**	*	*	*
	Cheng et al.	*	*	NA	*	**	**	*	*
	Crusius et al.	*	*	NA	*	**	*	*	*
	Ennas et al.	*	*	*	*	**	*	*	*
	Garcia-Gonzalez et al.	*	*	*	*	**	*	*	*
	Gu et al.	*	*	NA	*	**	*	*	*
	Gu et al.	*	*	NA	*	**	*	*	*
	Gunter et al.	*	*	NA	*	**	**	*	*
	Jeng et al.	*	*	*	*	**	*	*	*
	Lakhanpal et al.	*	*	NA	*	**	*	*	*
	Lan et al.	*	*	*	*	**	*	*	*
	Lee et al.	*	*	*	*	**	*	*	*
	Liu et al.	*	*	*	*	**	*	*	*
	Mahajan et al.	*	*	*	*	**	*	*	*
	Mou et al.	*	*	NA	*	**	*	*	*
	Niwa et al.	*	*	NA	*	**	*	*	*
	Niwa et al.	*	*	NA	*	**	*	*	*
	Purdue et al.	*	*	NA	*	**	*	*	*
	Tsai et al.	*	*	*	*	**	*	*	*
	Wang et al.	*	*	*	*	**	*	*	*
	Yri et al.	*	*	NA	*	**	*	*	*

A study can be awarded a maximum of one star (*) for each numbered item within the Selection and Exposure categories. A maximum of two stars (**) can be given for Comparability. Abbreviations: NA, not applicable.

### Quantitative synthesis

***rs1041981***

**Figure 2:**
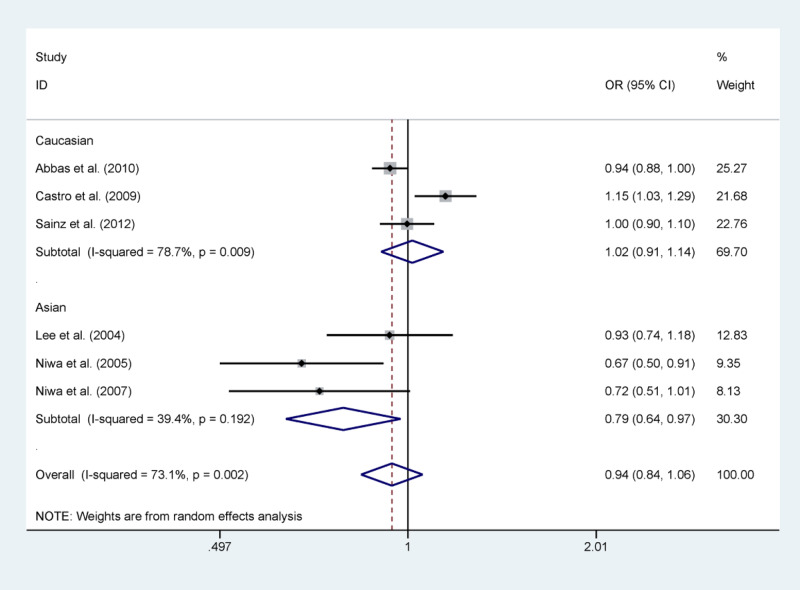
Forest plot of *LTA* rs1041981 polymorphism and cancer risk in allelic contrast stratified by ethnicity

The pooled results based on six included studies [11–16] (including 6427 cases and 9714 controls) indicated that no significant association between rs1041981 polymorphism and cancer risk was found. However, in the stratification analysis by ethnicity, we observed that Asian group was significantly related to a reduced risk of cancer in allelic contrast (B vs A: OR = 0.79, 95% confidence interval (CI) = 0.64–0.97, *P*=0.027, Figure 2) and dominant model (BB+AB vs AA: OR = 0.67, 95% CI = 0.52–0.87, *P*=0.002). Moreover, when the subgroup analysis was performed based on source of controls, hospital-based control group was significantly related to a decreased risk of cancer in allelic contrast (B vs A: OR = 0.69, 95% CI = 0.55–0.87, *P*=0.002) and dominant model (BB+AB vs AA: OR = 0.58, 95% CI = 0.42–0.78, *P*=0.000) (Table 3).

**Table 3:** Meta-analysis of rs1041981

Variables	*n*	Allelic contrast		Dominant model		Recessive model	
		*P*, OR (99% CI)	*P* (Q test), *I^2^*	*P*, OR (99% CI)	*P* (Q test), *I^2^*	*P*, OR (99% CI)	*P* (Q test), *I^2^*
Total	6	0.307, 0.94 (0.84, 1.06)	0.002, 73.1%	0.163, 0.89 (0.75, 1.05)	0.002, 73.1%	0.607, 1.03 (0.91, 1.17)	0.214, 29.4%
Ethnicity							
Asian	3	0.027, 0.79 (0.64, 0.97)	0.192, 39.4%	0.002, 0.67 (0.52, 0.87)	0.303, 16.2%	0.433, 0.87 (0.63, 1.22)	0.327, 10.5%
Caucasian	3	0.782, 1.02 (0.91, 1.14)	0.009, 78.7%	0.951, 1.01 (0.85, 1.18)	0.014, 76.4%	0.380, 1.06 (0.93, 1.22)	0.152, 47.0%
Source of control							
PB	4	0.926, 1.00 (0.91, 1.11)	0.022, 68.7%	0.788, 0.98 (0.85, 1.13)	0.029, 66.8%	0.329, 1.06 (0.95, 1.18)	0.287, 20.5%
HB	2	0.002, 0.69 (0.55, 0.87)	0.774, 0.0%	0.000, 0.58 (0.42, 0.78)	0.813, 0.0%	0.171, 0.72 (0.46, 1.15)	0.336, 0.0%

Abbreviations: HB, hospital-based; *n*, number; PB, population-based.

#### rs2229094

The pooled results based on four included studies [11,17–19] (including 6227 cases and 8562 controls) indicated that no significant association between rs2229094 polymorphism and cancer risk was found. Further subgroup analysis by ethnicity also indicated that no significant result was uncovered (Supplementary Table S1).

#### rs2239704

**Figure 3:**
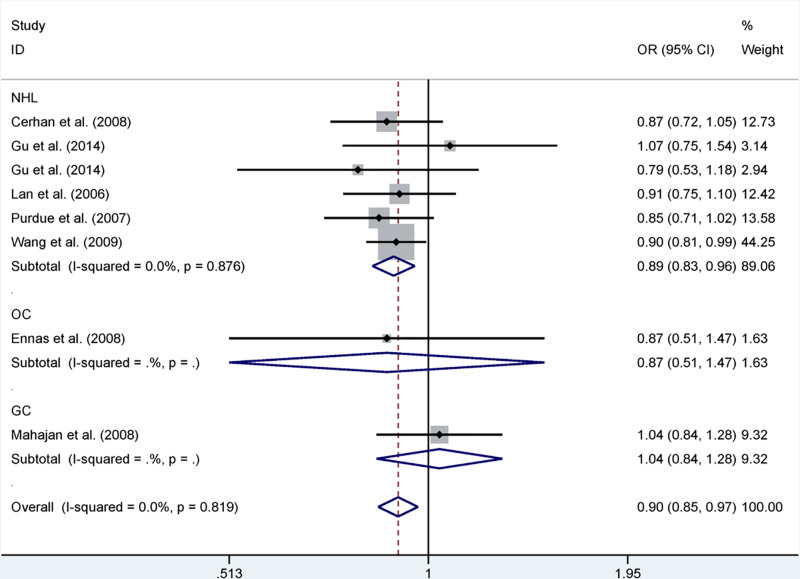
Forest plot of *LTA* rs2239704 polymorphism and cancer risk in allelic contrast stratified by cancer type Abbreviations: GC, gastric cancer; OC, other cancer.

The pooled results based on eight included studies [18–24] (including 3550 cases and 3962 controls) suggested that rs2239704 reduced the risk of cancer in allelic contrast (B vs A: OR = 0.90, 95% CI = 0.85–0.97, *P*=0.003), dominant model (BB+AB vs AA: OR = 0.88, 95% CI = 0.80–0.96, *P*=0.006) and recessive model (BB vs AA+AB: OR = 0.88, 95% CI = 0.77–0.99, *P*=0.040). Furthermore, in the stratification analysis by cancer type, we observed that rs2239704 reduced the risk of NHL in allelic contrast (B vs A: OR = 0.89, 95% CI = 0.83–0.96, *P*=0.001, Figure 3), dominant model (BB+AB vs AA: OR = 0.88, 95% CI = 0.80–0.97, *P*=0.011) and recessive model (BB vs AA+AB: OR = 0.83, 95% CI = 0.72–0.95, *P*=0.006). Moreover, when the subgroup analysis was performed based on ethnicity, source of control and genotyping, we found mixed ethnicity was significantly related to a reduced risk of cancer in allelic contrast (B vs A: OR = 0.89, 95% CI = 0.83–0.97, *P*=0.006), dominant model (BB+AB vs AA: OR = 0.89, 95% CI = 0.79–1.00, *P*=0.042) and recessive model (BB vs AA+AB: OR = 0.82, 95% CI = 0.71–0.96, *P*=0.013) (Table 4).

**Table 4:** Meta-analysis of rs2239704

Variables	*n*	Allele contrast		Dominant model		Recessive model	
		*P*, OR (99% CI)	*P* (Q test), *I^2^*	*P*, OR(99% CI)	*P* (Q test), *I^2^*	*P*, OR(99% CI)	*P* (Q test), *I^2^*
Total	8	0.003, 0.90 (0.85, 0.97)	0.819, 0.0%	0.006, 0.88 (0.80, 0.96)	0.831, 0.0%	0.040, 0.88 (0.77, 0.99)	0.444, 0.0%
Cancer type							
NHL	6	0.001, 0.89 (0.83, 0.96)	0.876, 0.0%	0.011, 0.88 (0.80, 0.97)	0.626, 0.0%	0.006, 0.83 (0.72, 0.95)	0.958, 0.0%
GC	1	0.733, 1.04 (0.84, 1.28)	NA	0.371, 0.86 (0.61, 1.20)	NA	0.128, 1.32 (0.92, 1.87)	NA
OC	1	0.605, 0.87 (0.51, 1.47)	NA	0.596, 0.81 (0.38, 1.75)	NA	0.778, 0.87 (0.34, 2.24)	NA
Ethnicity							
Asian	2	0.633, 0.94 (0.72, 1.22)	0.264, 19.8%	0.645, 0.92 (0.63, 1.33)	0.084, 66.5%	0.775, 0.93 (0.55, 1.55)	0.791, 0.0%
Caucasian	3	0.227, 0.92 (0.81, 1.05)	0.356, 3.3%	0.061, 0.83 (0.68, 1.01)	0.964, 0.0%	0.924, 1.01 (0.79, 1.29)	0.131, 50.8%
Mixed	3	0.006, 0.89 (0.83, 0.97)	0.944, 0.0%	0.042, 0.89 (0.79, 1.00)	0.978, 0.0%	0.013, 0.82 (0.71, 0.96)	0.698, 0.0%
Source of control							
PB	6	0.033, 0.92 (0.85, 0.99)	0.728, 0.0%	0.029, 0.88 (0.79, 0.99)	0.681, 0.0%	0.258, 0.92 (0.80, 1.06)	0.431, 0.0%
HB	2	0.022, 0.86 (0.75, 0.98)	0.849, 0.0%	0.095, 0.85 (0.71, 1.03)	0.581, 0.0%	0.029, 0.75 (0.58, 0.97)	0.710, 0.0%
Genotyping							
PCR	5	0.023, 0.91 (0.85, 0.99)	0.546, 0.0%	0.032, 0.89 (0.79, 0.99)	0.548, 0.0%	0.153, 0.90 (0.78, 1.04)	0.174, 37.0%
TaqMan	3	0.041, 0.88 (0.77, 0.99)	0.877, 0.0%	0.087, 0.85 (0.71, 1.02)	0.830, 0.0%	0.110, 0.82 (0.64, 1.05)	0.955, 0.0%

Abbreviations: GC, gastric cancer; HB, hospital-based; *n*, number; NA, not applicable; OC, other cancer; PB, population-based; PCR, polymerase chain reaction.

#### rs746868

The pooled results based on four included studies [18,25–27] (including 1351 cases and 2145 controls) indicated that no significant association between rs746868 polymorphism and risk of cancer was uncovered. Moreover, in the subgroup analysis by cancer type, ethnicity and source of control, similar results were found. (Supplementary Table S2).

#### rs909253

**Figure 4:**
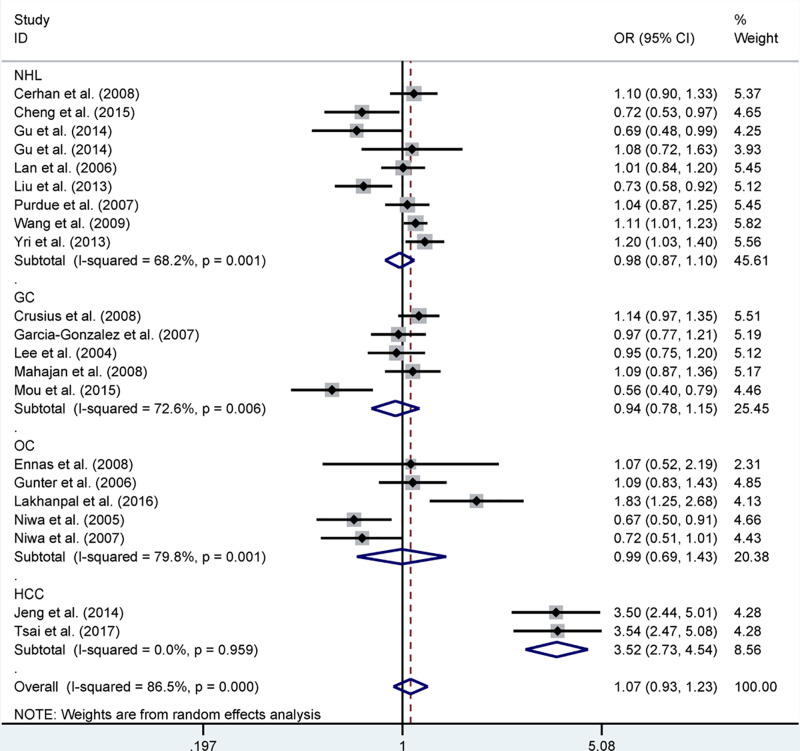
Forest plot of *LTA* rs909253 polymorphism and cancer risk in allelic contrast stratified by cancer type Abbreviations: GC, gastric cancer; HCC, hepatocellular carcinoma; OC, other cancer.

The pooled results based on 21 included studies [13–15,18–34] (including 7022 cases and 8968 controls) indicated that no significant association between rs909253 polymorphism and cancer risk was found. However, in the stratification analysis by cancer type, we observed that rs909253 polymorphism was significantly related to an increased risk of HCC in allelic contrast (B vs A: OR = 3.52, 95% CI = 2.73–4.54, *P*=0.000, Figure 4), dominant model (BB+AB vs AA: OR = 4.33, 95% CI = 3.07–6.09, *P*=0.000) and recessive model (BB vs AA+AB: OR = 4.92, 95% CI = 2.92–8.29, *P*=0.000). In addition, in the stratification analysis by ethnicity, we observed that rs909253 polymorphism was significantly related to an increased risk of Caucasian ethnicity in allelic contrast (B vs A: OR = 1.10, 95% CI = 1.02–1.20, *P*=0.019), and mixed ethnicity in allelic contrast (B vs A: OR = 1.09, 95% CI = 1.01–1.17, *P*=0.024), dominant model (BB+AB vs AA: OR = 1.11, 95% CI = 1.01–1.23, *P*=0.039). Moreover, in the stratification analysis by source of control, genotyping and HWE, null result was found (Table 5).

**Table 5:** Meta-analysis of rs909253

Variables	*n*	Allele contrast		Dominant model		Recessive model	
		*P*, OR (99% CI)	*P* (Q test), *I^2^*	*P*, OR(99% CI)	*P* (Q test), *I^2^*	*P*, OR(99% CI)	*P* (Q test), *I^2^*
Total	21	0.349, 1.07 (0.93, 1.23)	0.000, 86.5%	0.198, 1.12 (0.94, 1.34)	0.000, 84.2%	0.993, 1.00 (0.81, 1.23)	0.000, 73.9%
Cancer type							
NHL	9	0.717, 0.98 (0.87, 1.10)	0.001, 68.2%	0.599, 1.04 (0.91, 1.18)	0.056, 47.3%	0.360, 0.90 (0.71, 1.13)	0.004, 64.2%
GC	5	0.567, 0.94 (0.78, 1.15)	0.006, 72.6%	0.775, 0.96 (0.74, 1.25)	0.007, 71.6%	0.511, 0.87 (0.57, 1.32)	0.005, 73.4%
HCC	2	0.000, 3.52 (2.73, 4.54)	0.959, 0.0%	0.000, 4.33 (3.07, 6.09)	0.928, 0.0%	0.000, 4.92 (2.92, 8.29)	1.000, 0.0%
OC	5	0.968, 0.99 (0.69, 1.43)	0.001, 79.8%	0.759, 1.11 (0.58, 2.13)	0.000, 87.7%	0.638, 0.93 (0.70, 1.25)	0.555, 0.0%
Ethnicity							
Asian	11	0.707, 1.07 (0.75, 1.54)	0.000, 92.9%	0.507, 1.17 (0.74, 1.86)	0.000, 91.3%	0.753, 0.92 (0.56, 1.52)	0.000, 85.4%
Caucasian	6	0.019, 1.10 (1.02, 1.20)	0.697, 0.0%	0.064, 1.15 (0.99, 1.34)	0.141, 39.6%	0.470, 1.07 (0.90, 1.27)	0.534, 0.0%
Mixed	4	0.024, 1.09 (1.01, 1.17)	0.860, 0.0%	0.039, 1.11 (1.01, 1.23)	0.758, 0.0%	0.136, 1.12 (0.96, 1.31)	0.984, 0.0%
Source of control							
PB	13	0.066, 1.18 (0.99, 1.42)	0.000, 88.4%	0.069, 1.23 (0.98, 1.53)	0.000, 85.4%	0.225, 1.18 (0.90, 1.53)	0.000, 75.7%
HB	8	0.370, 0.91 (0.73, 1.12)	0.000, 80.3%	0.858, 0.97 (0.71, 1.34)	0.000, 81.8%	0.119, 0.76 (0.54, 1.07)	0.003, 67.3%
Genotyping							
PCR	18	0.377, 1.08 (0.91, 1.27)	0.000, 88.5%	0.223, 1.14 (0.92, 1.40)	0.000, 86.5%	0.996, 1.00 (0.78, 1.27)	0.000, 77.7%
TaqMan	3	0.706, 1.02 (0.90, 1.16)	0.957, 0.0%	0.649, 1.04 (0.88, 1.23)	0.846, 0.0%	0.929, 1.01 (0.78, 1.31)	0.927, 0.0%
HWE							
Y	19	0.304, 1.08 (0.94, 1.24)	0.000, 85.8%	0.292, 1.10 (0.92, 1.30)	0.000, 82.5%	0.643, 1.05 (0.85, 1.29)	0.000, 71.5%
N	2	0.985, 1.01 (0.32, 3.21)	0.000, 95.2%	0.592, 1.72 (0.24, 12.6)	0.000, 95.8%	0.403, 0.58 (0.16, 2.10)	0.003, 88.6%

Abbreviations: GC, gastric cancer; HB, hospital-based; HCC, hepatocellular carcinoma; *n*, number; N, no; OC, other cancer; PB, population-based; PCR, polymerase chain reaction; Y, yes.

### Sensitivity analysis and publication bias

Sensitivity analysis were performed to evaluate the influence of each separate case–control study. The results showed that there was no material alteration in corresponding pooled ORs for rs1041981, rs2229094, rs2239704, rs746868, rs909253 (Supplementary Figures S1–S5). In addition, Begg’s test and Egger’s regression test were performed to evaluate the publication bias. As for rs1041981, rs2229094, rs2239704, rs746868 and rs909253, no evidence of publication bias was identified (Supplementary Table S3).

### TSA

**Figure 5:**
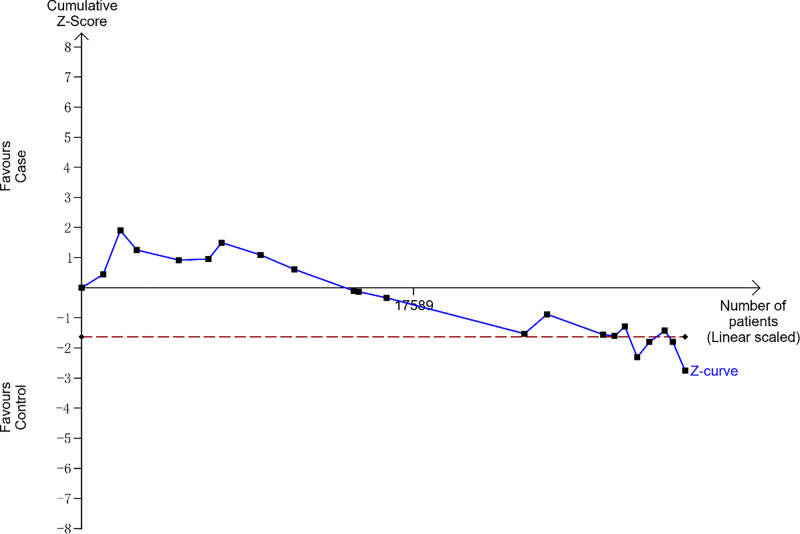
TSA for *LTA* rs909253 polymorphism under the allele contrast model

To evaluate random errors, we performed TSA (Figure 5). This analysis showed that the cumulative z-curve did not cross the trial sequential monitoring boundary and the required information size, suggesting that more evidences are needed to verify the conclusions.

## Discussion

In the present study, a total of 24 articles including 43 case–control studies were enrolled to validate the association between five *LTA* gene polymorphisms (rs1041981, rs2229094, rs2239704, rs746868, rs909253) and the risk of cancer. We identified that rs2239704 was inversely associated with the risk of cancer under different genetic models. However, for *LTA* rs1041981, rs2229094, rs746868, rs909253 polymorphisms, no significant association with cancer risk was uncovered.

In subgroup meta-analysis stratified by cancer type, we found that rs2239704 was significantly reduced NHL susceptibility. Huang et al. reported rs2239704 polymorphism was correlated with cancer and positive association in North Americans [35]. However, they included studies that contained buccal samples for SNP analysis or insufficient data studies [37–39]. We strictly follow the inclusion and exclusion criteria to include the literature. And our results indicated that rs2239704 was significantly reduced cancer susceptibility in mixed ethnicity, hospital-based control and polymerase chain reaction (PCR) genotyping subgroups. Despite of several possible bias, we still could conclude that rs2239704 could reduce cancer susceptibility.

In the stratified analysis of rs1041981, we found that Asians might have less susceptibility to cancer. Unlike the study by Huang et al. [35], we excluded two studies, one of which was autopsy specimen for SNP analysis [10] and the other was a study of HIV-infected patients [40]. The literature thus incorporated has a better baseline consistency and is more reflective of the real situation. Our results were consistent with the results of Huang et al.[35]. Due to the small sample size, we were unable to evaluate the role of rs1041981 in Caucasians. Larger sample size studies are needed for further evaluation. However, based on the current studies, we might conclude that rs1041981 could reduce cancer susceptibility in Asians.

For *LTA* rs2229094 and rs746848, only four studies reported their relationship with cancer in each group. No significant results were found. Huang et al. reported positive association between rs2229094 and cancer risk [35], which could be the bias from report by Takei et al. [10]. Because of the small sample size, we could not draw any conclusions based on current literature.

Although the overall analysis of rs909253 indicated a null result for cancer risk, the risk of cancer for Caucasians and HCC susceptibility were significantly increased in the stratified analysis by ethnicity and cancer types. In addition, some of the control groups did not match HWE, we can not exclude the possibility that may cause the bias. Then, subgroup analysis by HWE showed that HWE status did not cause the bias of results. Huang et al. did not report the relationship of rs909253 and cancer risk, because it might be present in high linkage disequilibrium with other four SNPs [8,9]. However, our results identified that the function of rs909253 was opposite to rs2239704 and rs1041981. So, further studies with larger sample size are required to identify the role of *LTA* rs909253 and the linkage disequilibrium with other SNPs.

In the present study, we have put great effort on carefully searching for eligible studies. In order to obtain more accurate and reliable results, we conducted a comprehensive search to verify more eligible studies. Then, we used NOS to evaluate the quality of the included studies, eliminate low-quality studies and improve overall research quality. In order to provide the sources of heterogeneity, subgroup analysis was performed by ethnicity, cancer type, source of controls, genotyping and so on. In addition, sensitivity analysis was used to confirm the stability of the studies. Egger’s and Begg’s tests were used to assess publication bias. However, several limitations in our study should be noted. First, small sample size limits the reliability of the results for some polymorphisms. Second, we just included the studies published in English, which may influence the effects of the polymorphisms. Third, we mainly evaluated the relationship between *LTA* polymorphisms with various cancers, and we could not get enough data for some cancer types. Fourth, we did not assess the linkage disequilibrium, which might not reflect the real function correctly. In future, more well-designed case–control studies are needed to investigate the functions of LTA polymorphisms.

## Conclusion

Our meta-analysis suggests that *LTA* rs2239704 polymorphism is inversely associated with the risk of cancer, as is *LTA* rs1041981 polymorphism in Asia. While, LTA rs909253 polymorphism is a risk factor for HCC in Caucasians. Further studies with larger sample size are needed to confirm these findings.

## Supplementary Material

Supplementary Figures S1-S5 and Tables S1-S3

## Abbreviations

CI: confidence interval
HWE: Hardy–Weinberg equilibrium
LTA: lymphotoxin-α
NHL: non-Hodgkin lymphoma
NOS: Newcastle–Ottawa scale
OR: odds ratio
SNP: single nucleic polymorphism
TNF: tumor necrosis factor
TSA: trial sequential analysis
