# Supplementary material for: The effect of LTA gene polymorphisms on cancer risk: an updated systematic review and meta- analysis
Source: Biosci Rep. 2020 May 28;40(5):BSR20192320. doi: 10.1042/BSR20192320 (PMC7256675; doi:10.1042/BSR20192320)
Supplement: Supplementary Figures S1-S5 and Tables S1-S3 [file BSR-2019-2320_supp.pdf]

Supplementary figure 1. Sensitivity analysis of *LTA* rs1041981.

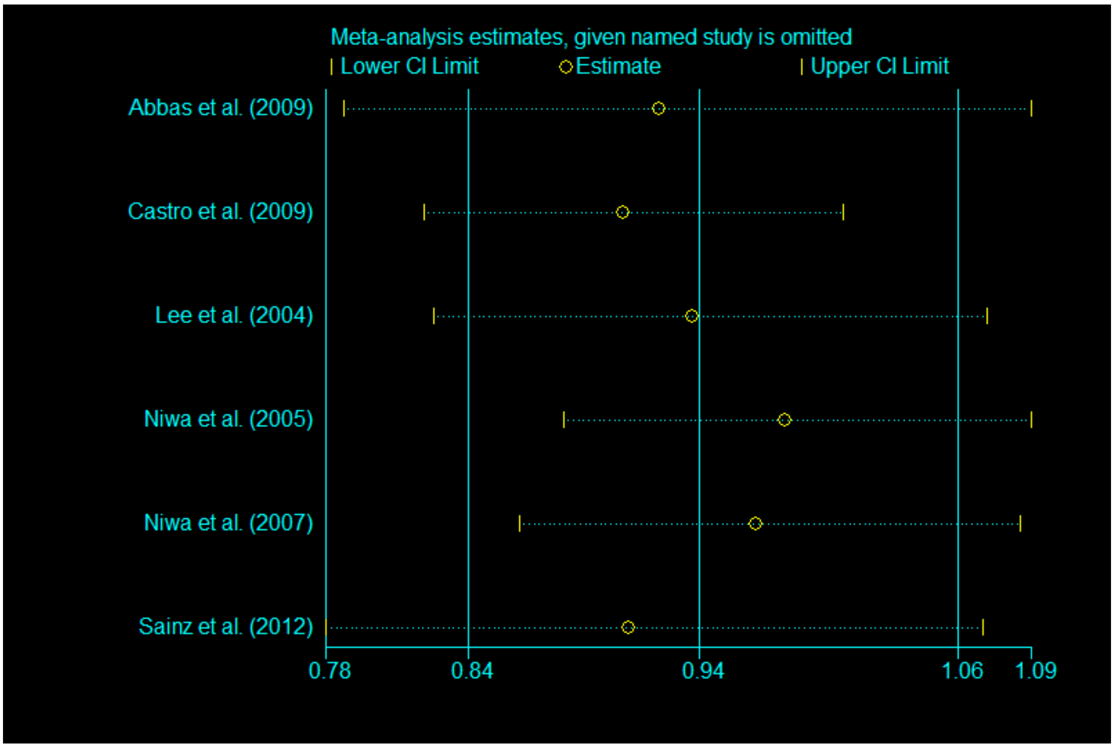

Supplementary figure 2. Sensitivity analysis of *LTA* rs2229094.

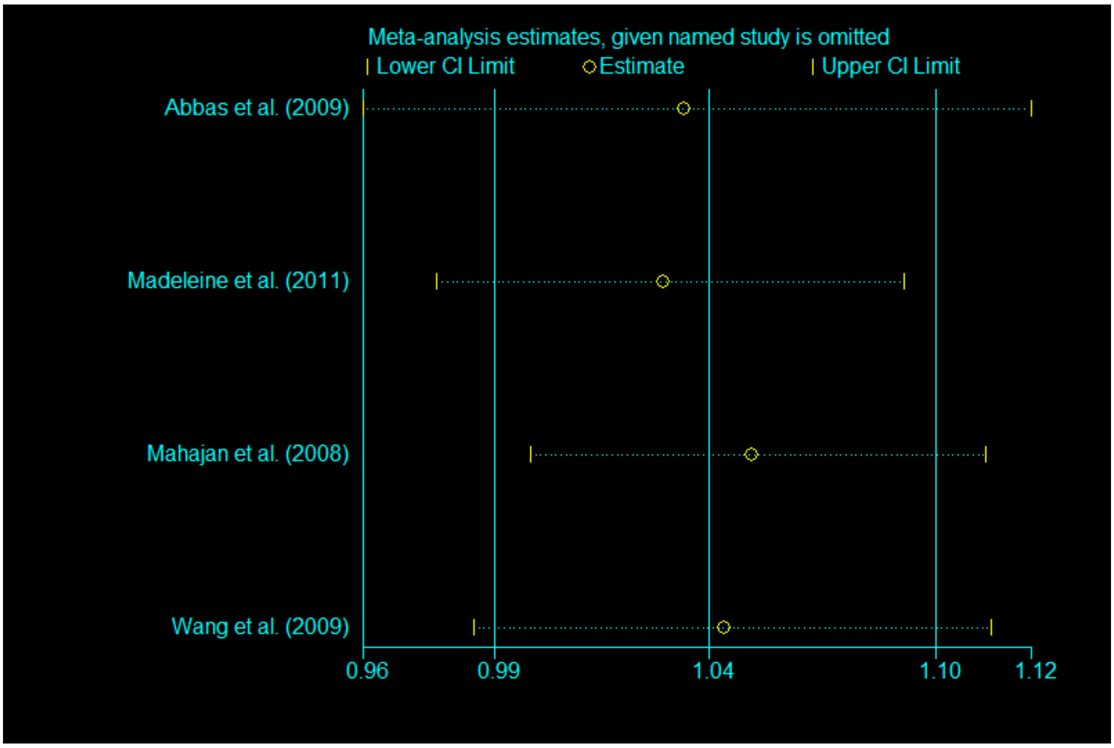

Supplementary figure 3. Sensitivity analysis of *LTA* rs2239704.

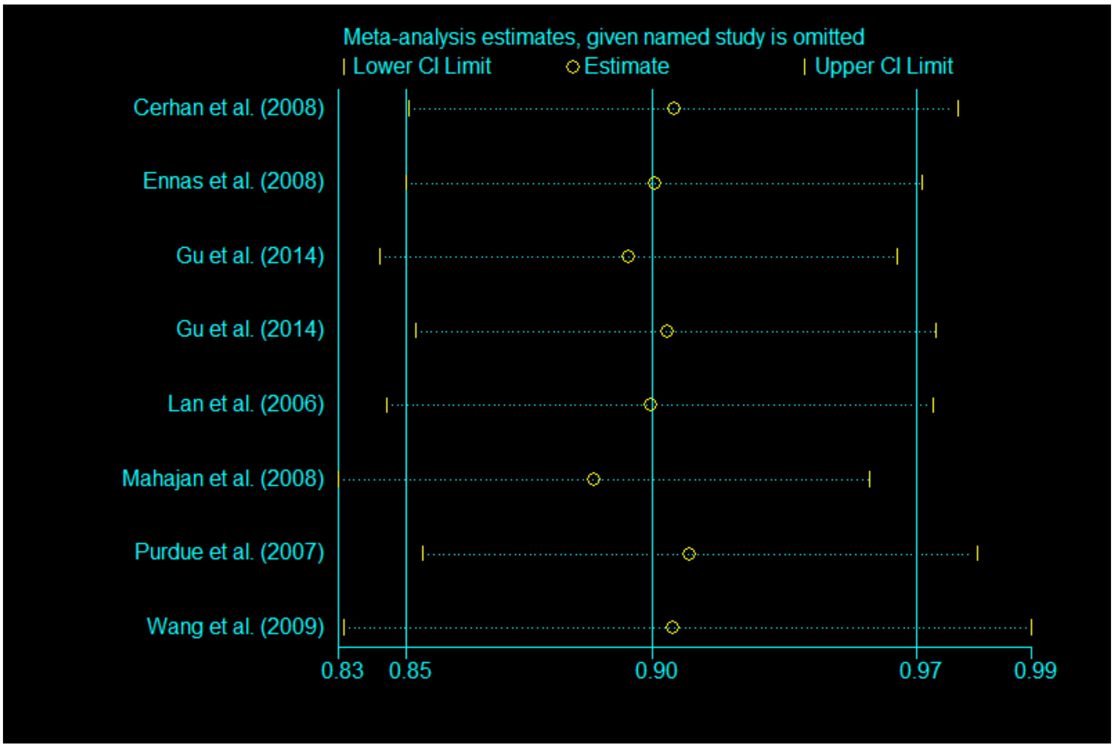

Supplementary figure 4. Sensitivity analysis of *LTA* rs746868.

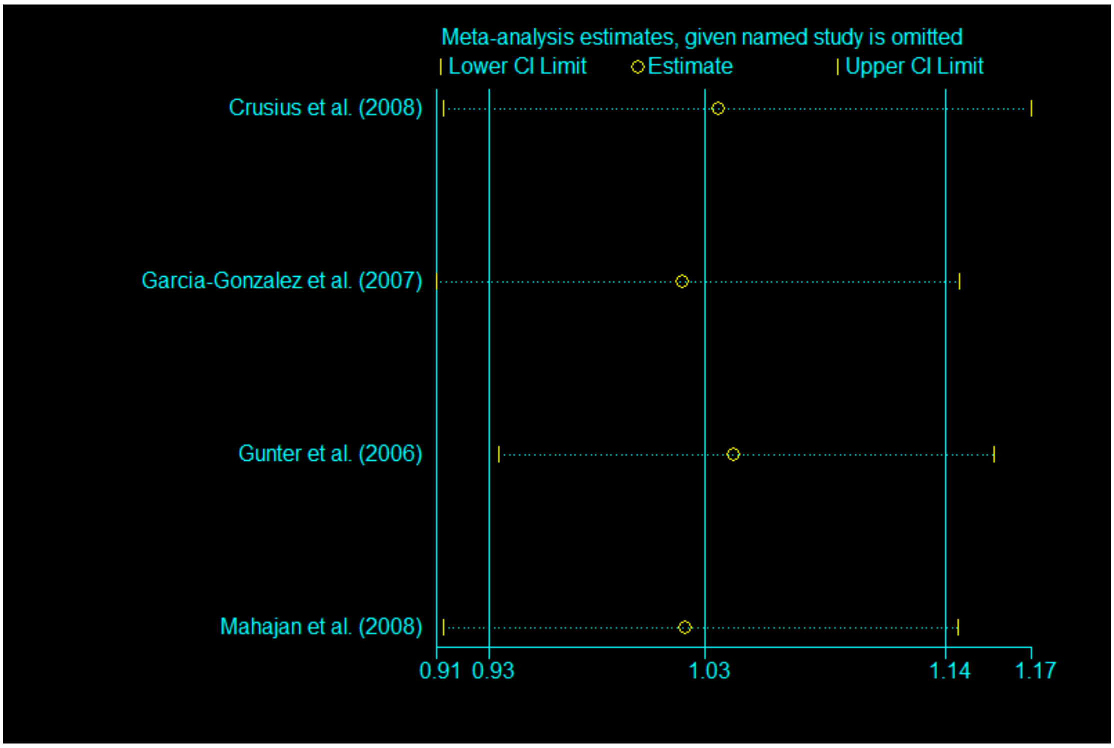

Supplementary figure 5. Sensitivity analysis of *LTA* rs909253.

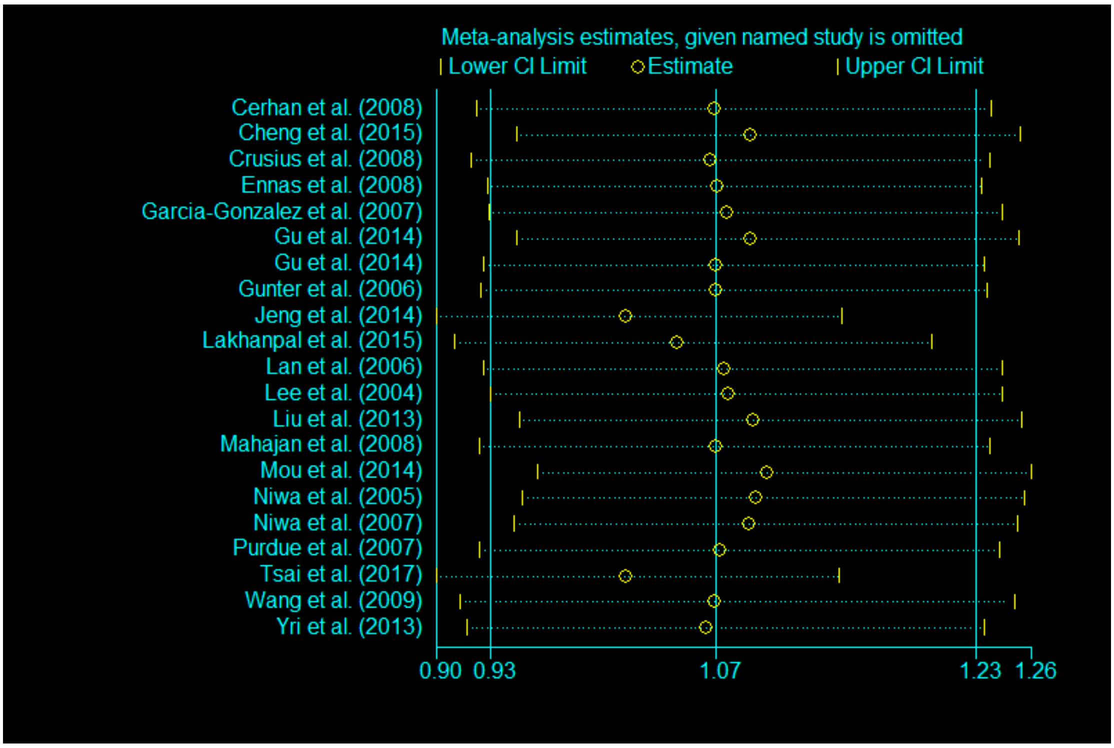

Supplementary Table 1.Meta-analysis of rs2229094.

Variables	n	Allele model		Dominant model		Recessive model	
		p, OR(99% CI)	*p* (Q test), I^2	*p*, OR(99% CI)	*p* (Q test), I^2	*p*, OR(99% CI)	*p* (Q test), I^2
Total	4	0.121, 1.04(0.99, 1.10)	0.244, 28.0%	0.806, 1.01(0.94, 1.08)	0.067, 58.1%	0.001, 1.24(1.09, 1.40)	0.623, 0.0%
Ethnicity							
Caucasian	2	0.380, 1.03(0.96, 1.10)	0.084, 66.4%	0.725, 0.98(0.90, 1.07)	0.015, 83.2%	0.005, 1.26(1.07, 1.49)	0.399, 0.0%
Mixed	2	0.165, 1.06(0.98, 1.16)	0.342, 0.0%	0.403, 1.05(0.94, 1.16)	0.495, 0.0%	0.085, 1.20(0.98, 1.47)	0.348, 0.0%

n: number; OR: odds ratio; CI: confidence interval.

Supplementary Table 2.Meta-analysis of rs746868.

Variables	n	Allele contrast		Dominant model		Recessive model	
		p, OR(99% CI)	*p* (Q test), I^2	*p*, OR(99% CI)	*p* (Q test), I^2	*p*, OR(99% CI)	*p* (Q test), I^2
Total	4	0.599, 1.03(0.93, 1.14)	0.918, 0.0%	0.972, 1.00(0.86, 1.16)	0.898, 0.0%	0.313, 1.10(0.92, 1.32)	0.768, 0.0%
Cancer type							
GC	3	0.476, 1.04(0.93, 1.16)	0.940, 0.0%	0.911, 1.01(0.86, 1.18)	0.800, 0.0%	0.249, 1.12(0.92, 1.36)	0.685, 0.0%
OC	1	0.698, 0.95(0.72, 1.25)	NA	0.712, 0.93(0.63, 1.38)	NA	0.795, 0.93(0.52, 1.64)	NA
Ethnicity							
Caucasian	3	0.476, 1.04(0.93, 1.16)	0.940, 0.0%	0.911, 1.01(0.86, 1.18)	0.800, 0.0%	0.249, 1.12(0.92, 1.36)	0.685, 0.0%
Mixed	1	0.698, 0.95(0.72, 1.25)	NA	0.712, 0.93(0.63, 1.38)	NA	0.795, 0.93(0.52, 1.64)	NA
Source of control							
PB	3	0.476, 1.04(0.93, 1.16)	0.940, 0.0%	0.911, 1.01(0.86, 1.18)	0.800, 0.0%	0.249, 1.12(0.92, 1.36)	0.685, 0.0%
HB	1	0.698, 0.95(0.72, 1.25)	NA	0.712, 0.93(0.63, 1.38)	NA	0.795, 0.93(0.52, 1.64)	NA

n: number; GC: gastric cancer; OC: other cancer; PB: population-based; HB: hospital-based; OR: odds ratio; CI: confidence interval; NA: not applicable.

Supplementary Table 3.Publication bias of the five polymorphisms of LTA.

Variables	Allelic contrast (*p*)	Dominant model (*p*)	Recessive model (*p*)
rs1041981			
Egger's test	0.445	0.949	0.468
Begg's test	0.452	0.764	0.260
rs2229094			
Egger's test	0.525	0.432	0.357
Begg's test	0.734	0.734	0.308
rs2239704			
Egger's test	0.786	0.857	0.824
Begg's test	0.536	0.711	0.902
rs746868			
Egger's test	0.538	0.440	0.623
Begg's test	0.734	0.734	0.734
rs909253			
Egger's test	0.956	0.875	0.578
Begg's test	0.349	0.566	0.305
